# Role of pharmacoepidemiology studies in addressing pharmacovigilance questions: a case example of pancreatitis risk among ulcerative colitis patients using mesalazine

**DOI:** 10.1007/s00228-014-1660-7

**Published:** 2014-03-11

**Authors:** Leo Russo, Gary Schneider, Margarita Hauser Gardiner, Stephan Lanes, Paul Streck, Susan Rosen

**Affiliations:** 1 Shire Development LLC, Wayne, PA USA; 2 Evidera, Lexington, MA USA; 3 Department of Health Economics, Outcomes Research and Epidemiology, Shire Research and Development, 725 Chesterbrook Boulevard, Wayne, PA 19087 USA

**Keywords:** Pancreatitis, Ulcerative colitis, Mesalazine, Pharmacoepidemiology, Pharmacovigilance

## Abstract

**Purpose:**

Well-designed pharmacoepidemiology studies address several limitations of postmarketing spontaneous reports in regard to signal evaluation. This study evaluated a signal of disproportionate reporting of acute pancreatitis cases observed in patients with ulcerative colitis (UC) treated with MMX Multi Matrix System® (MMX®) mesalazine and demonstrated how inherent limitations of postmarketing reports were overcome.

**Methods:**

Adults with UC who were new users of MMX mesalazine or another branded mesalazine (controlled-release, delayed-release, or extended-release mesalazine; balsalazide disodium; olsalazine sodium; sulfasalazine; or sulfasalazine delayed-release) were identified from a large US administrative healthcare claims database. Acute pancreatitis incidence rates were compared between patients on MMX mesalazine versus comparator therapies. Propensity scores were used to match patients on MMX mesalazine with patients on comparator drugs to achieve a balance of baseline patient factors.

**Results:**

Crude incidence rates [95 % confidence interval (CI)] of acute pancreatitis among patients on MMX mesalazine were similar to those of patients on comparator therapies [8.55 (5.54–13.21) vs 10.05 (7.54–13.41) per 1000 person-years]; the resulting incidence rate ratio (IRR) was [0.85 (0.48–1.47)]. Propensity score-matching had little influence on the IRR [0.84 (0.46–1.55)]; nor did further adjustment by demographic characteristics, daily dose, and causes of acute pancreatitis [0.76 (0.41–1.43)].

**Conclusion:**

Findings of no increase in pancreatitis risk with MMX mesalazine demonstrate the value of pharmacoepidemiology studies for evaluating a drug’s postmarket safety profile when confronted with spontaneous reporting data suggestive of a safety issue.

**Electronic supplementary material:**

The online version of this article (doi:10.1007/s00228-014-1660-7) contains supplementary material, which is available to authorized users.

## Introduction

Given limited sample sizes, short duration, and lack of generalizability of preapproval clinical trials, a drug’s safety profile at the time of regulatory approval is often incomplete. Therefore, postmarketing safety surveillance is an essential exercise in detecting and evaluating adverse drug reactions (ADRs) of a given drug [1]. The primary source of postmarket safety surveillance data is voluntary reporting by clinicians and consumers [2]. Although now computerized, the spontaneous reporting system has been in place in the United States since the 1950s [3]. Strengths of the system include the potential to encapsulate rich clinical details on a single case and the ability to detect rare ADRs (<1:1,000) compared with clinical trials [3]. However, limitations of the system are well recognized and include incomplete reporting, the presence of reporting biases that may result in both under- and overascertainment, the lack of a denominator for balanced comparisons, and the inability to determine incidence rates (IRs) from spontaneous reports [3].

In this paper, we present an example of how disproportionate reporting of pancreatitis cases associated with the postmarket surveillance of MMX Multi Matrix System®^,^ 1(MMX®^,1^) mesalazine, a 5-aminosalicylic acid (5-ASA) formulation, was further investigated using a pharmacoepidemiology study. Treatment with 5-ASA is considered first-line therapy for patients with active mild to moderate ulcerative colitis (UC) [4]. MMX mesalazine is a once-daily formulation of 5-ASA administered orally that is effective in induction and maintenance of UC remission [5–8]. Mesalazine is also available in the United States in several other formulations (i.e., controlled-release mesalazine, delayed-release mesalazine, extended-release mesalazine, balsalazide disodium, olsalazine sodium, sulfasalazine, or sulfasalazine delayed release).

Acute pancreatitis is an inflammatory disease of the pancreas characterized by severe acute upper abdominal pain and elevated blood levels of serum amylase and/or lipase [9]. Annual incidence of acute pancreatitis is estimated to be 32 cases per 100,000 persons in the general population [10]. Although rare, pancreatitis is an event associated with UC as well as with mesalazine treatment of UC [11, 12]. As such, pancreatitis events were observed during clinical development and postmarketing experience of MMX mesalazine, and this class effect is listed in its US package insert [13]. Analysis of reports received by the manufacturer and entered into the postmarketing surveillance database revealed a higher rate of reporting for pancreatitis with MMX mesalazine (21.0 cases per 100,000 person-years of exposure) compared with controlled-release mesalazine (1.3 cases per 100,000 person-years) [data on file]. In addition, the US Food and Drug Administration (FDA) Adverse Event Reporting System Database [14] also revealed this pattern of disproportionate reporting. However, due to the aforementioned reporting biases, the large differences in the time these drugs have been on the US market (MMX mesalazine launch in 2007, controlled-release mesalazine in 1993, and delayed-release mesalazine in 1992) and other limitations in spontaneous reporting systems, further investigation was undertaken to determine whether the reporting data indicated a true association of pancreatitis with MMX mesalazine compared with other mesalazine formulations. A summary of the findings from the postmarketing spontaneous reporting data and the associated limitations in interpretation is presented in Table 1.

**Table 1:** Postmarketing spontaneous reporting data for pancreatitis with MMX® mesalazine

Findings from spontaneous reporting systems	
Shire Global Safety System^*a*^ [data on file]	• Higher rate of reporting for pancreatitis with MMX mesalazine (21.0 cases per 100,000 person-years of exposure) compared with another marketed controlled-release mesalazine formulation (controlled-release mesalazine; 1.3 cases per 100,000 person-years)
US FDA Adverse Event Reporting System (AERS) database [14]	• Disproportionate reporting of pancreatitis for MMX mesalazine compared with controlled-release mesalazine and delayed-release mesalazine [lower bound of the 95 % confidence interval of the Empirical Bayesian Geometric Mean (EB05) of pancreatitis cases: 5.78 for MMX mesalazine compared with 2.05 for controlled-release mesalazine and 1.92 for delayed-release mesalazine]
Limitations in interpretation [2, 3]	
	• Reporting biases due to the difference in time on the US market of the compared drugs (MMX mesalazine launch in 2007, controlled-release mesalazine in 1993, and delayed-release mesalazine in 1992)
	• Underreporting overall and differential underascertainment between drugs
	• Source population (denominator) is unknown, resulting in inability to calculate true incidence rates
	• Incomplete reporting of patient covariates required to adjust for confounding
	• Incomplete supporting data to validate or adjudicate endpoints

*FDA* Food and Drug Administration

^a^Fully validated safety database utilized for data entry, storage, and analysis of adverse event information received from sources including but not limited to healthcare practitioners, regulatory authorities, clinical trials, consumers, and the published medical literature. The database allows for aggregate report production and reporting of individual-case safety reports to regulatory authorities

One strategy to investigate whether spontaneous ADR reports reflect a true association in the rate of pancreatitis with MMX mesalazine is to conduct a pharmacoepidemiology study with healthcare claims data [2]. The use of electronic healthcare data to monitor drug safety has evolved in recent years, as evidenced by the FDA’s Sentinel Initiative, a long-term program designed to build and implement a national electronic system for monitoring the safety of FDA-approved drugs [15]. The purpose of this paper is to describe a pharmacoepidemiology study designed to evaluate a signal observed in spontaneous reporting data for acute pancreatitis in patients with UC using MMX mesalazine, and to report on the methods and findings of this study and how it overcame the limitations of the postmarketing reports.

## Methods

### Study design

This retrospective cohort study analyzed US pharmacy and medical claims data from the Thomson Reuters (Truven Health) MarketScan® Commercial and Medicare supplemental claims databases. They are fully integrated patient-level databases covering healthcare delivery from the inpatient, outpatient, drug, and laboratory settings, as well as health risk assessment, and benefit design information from US commercially insured and Medicare supplemental populations. The MarketScan databases are compliant with the Health Insurance Portability and Accountability Act of 1996. Patient data provided by Truven Health were de-identified; therefore, per International Society for Pharmacoepidemiology Guidelines on Data Privacy, Medical Record Confidentiality, and Research in the Interest of Public Health [16], no Internal Review Board approval or patient authorization was required.

Using these databases, patient and treatment information was obtained for all patients with any diagnosis of UC [*International Classification of Diseases*, 9th revision, (ICD-9) 556, 556.0, 556.1, 556.5, 556.6, 556.8, and 556.9] between 1 July 2007 and 1 December 2010. Given the objective of this study, MMX mesalazine was considered the treatment of primary interest. Comparator drugs consisted of all other branded orally administered mesalazine-containing medications indicated for UC. Comparator 5-ASA UC drugs were combined into one category, which was subdivided into categories according to the specific mechanism of 5-ASA release: moisture dependent (controlled-release mesalazine), pH-dependent (delayed- and extended-release mesalazine), and azoreductase dependent (balsalazide disodium, olsalazine sodium, sulfasalazine, and sulfasalazine delayed release). The comparator drugs were restricted to branded, orally administered UC medications; suppositories and generic forms were not used as comparators. The rationale for these restrictions was to minimize potential bias when comparing MMX mesalazine (available as branded and for oral administration only) due to channelling of patients to older and less expensive UC drugs. The baseline period was defined as the 6-month interval prior to the initial dispensing of one of the study drugs. Follow-up began on the index date, defined as the first day of initial dispensing of a study drug after the 6-month drug-free interval, and continued until treatment discontinuation of index mesalazine treatment, date of first pancreatitis event, disenrollment from the database, or the end of the study period (31 December 2010).

The main study outcome was acute pancreatitis (ICD-9: 577.0) occurring within the exposure episode, which was defined as the period from the index date through 30 days following the days’ supply of the last prescription. Discontinuation was defined by determining the days between each consecutive pair of prescriptions; if this timeframe was longer than the days’ supply plus 30 days, then the patient was considered to have discontinued the index drug. Additionally, after 30 days from the days’ supply of the last prescription of the index drug, the patient was also defined as discontinued. As patient follow-up was censored in parallel with index drug discontinuation, there was no risk of any patient having exposure to multiple study drugs during follow-up. Per algorithms applied in prior database studies, acute pancreatitis must have been associated with a hospital/emergency room (ER) visit [17, 18]. Based on the claims-based algorithm in another study that produced the highest positive predictive value (82.2 %) for acute pancreatitis, if both chronic and acute pancreatitis were recorded in the same admission/ER visit, the event was assumed to be chronic pancreatitis [18]. Patients were censored from further follow-up after acute pancreatitis was diagnosed.

### Patient population

Eligible participants in this study were adults aged ≥18 years diagnosed with UC within 6 months prior to receiving one of the study drugs. Patients were required to be new users of MMX mesalazine or a comparator 5-ASA UC treatment and were categorized by the first mesalazine drug received. New users were defined as patients with one prescription or more for a study drug, with no prior use of MMX mesalazine or any of the comparator UC treatments in the previous 6 months, ensuring that the patient drug cohorts were mutually exclusive. The inclusion criteria used to identify patients were aligned with patient selection criteria in prior retrospective UC database studies [19–22]. Patient eligibility criteria for this analysis are provided in the Supplementary Table 1. To control for confounding by indication, analyses were also conducted on a propensity-matched set, wherein each patient from the MMX mesalazine cohort was matched one to one with a comparator patient by decile of propensity scores (i.e., the probability of being prescribed MMX mesalazine versus comparator, regardless of actual drug received). MMX mesalazine users not matched to a comparator were excluded from the propensity-matched set.

### Statistical analyses

Patient identification and the majority of analyses were conducted using SÆfetyWorks® software (UBC Corporation, Harrisburg, PA, USA). Data were processed into a common data model format prior to being loaded into the SÆfetyWorks environment. Because of limitations inherent in the SÆfetyWorks software, certain analyses (e.g., calculations and model building involving dose, conducted only for MMX mesalazine patients and comparators remaining after propensity-score matching) were conducted outside of the SÆfetyWorks platform using SAS, version 9.2 (SAS Institute Inc., Cary, NC, USA).

Unadjusted IRs were calculated as the number of acute pancreatitis cases per 1,000 person-years of follow-up; unadjusted IR ratios (IRRs) were calculated as the ratio of the MMX mesalazine IR to the comparator IR. As a measure of relative risk, an IRR <1.0 depicts lower incidence among patients receiving MMX mesalazine. MMX mesalazine IRs and related IRRs were further stratified individually by age and sex categories.

Prior to propensity-score matching, several sensitivity analyses were conducted by varying both the gap between refills (i.e., drug-persistence gap) and length of the risk period after the last treatment episode (interval at the end of the supply of the last prescription, within which a pancreatitis event would still be included in the study). These analyses were performed at 15, 30, and 60 days to evaluate the impact of assumptions about continuous treatment and the risk period to the unadjusted IRs. An explanation of propensity score methodology is provided in Supplementary Table 2.

Adjusted IRRs were estimated from the propensity-matched data set using a Poisson regression model, with the log of follow-up time as an offset parameter. To achieve balance in baseline risk, each patient in the MMX mesalazine cohort was matched to a comparator by propensity scores within the same decile of the propensity-score-distribution. After matching, IRR analyses comparing MMX mesalazine to comparators were conducted without further adjustment (i.e., propensity-score–matched only) and also using two further adjusted models (to control for possible residual confounding). The first adjusted model incorporated age group (18–29, 30–39, 40–49, 50+), gender, and continuous average daily dose measure. The second adjusted model also incorporated risk factors of acute pancreatitis, consistent with Frossard et al. [23]. Of note, not all variables were included in the final model; ones that were weakly associated in unadjusted models (*P* > 0.5) and collinear variables were excluded. Ultimately, only six of 49 possible variables were in final models. Propensity-matched sets were used for all adjusted analyses, whereas the entire study sample was used for unadjusted (crude) analyses.

For MMX mesalazine and comparators, the dose of each prescription was calculated on a per-patient basis as the number of pills dispensed multiplied by the medication strength in the propensity-matched set only. MMX mesalazine and comparators delayed-release, controlled-release, and extended-release mesalazine, as well as olsalazine, were considered to be in equivalent units of mesalazine. Mesalazine conversion factors were used to equate doses of balsalazide and sulfasalazine with the other mesalazines: 6,750 mg balsalazide and 6,200 mg sulfasalazine were considered roughly equivalent to 2,400 mg mesalazine [24, 25]. Although it was later discovered that the conversion for sulfasalazine should have been 6,000 mg (not 6,200 mg) as equivalent to 2,400 mg mesalazine, this small error affected 4 % of all comparators and was deemed not to have had a meaningful impact on dose classification. After application of the mesalazine conversion factors, the average daily dose per treatment course for each patient was calculated as the cumulative dose divided by the total days of supply, and average daily doses were categorized as low (<1,500 mg/day), medium (1,500–4,800 mg/day), or high (>4,800 mg/day).

## Results

### Patient characteristics

A total of 14,936 patients (54 % male) were identified in the study. Patient demographic and baseline characteristics are summarized in Table 2. Mean age of the study sample was 46.5 years. Nine (0.06 %) patients were given prescriptions for both MMX mesalazine and a comparator mesalazine on their index date. These patients were included in the total cohort but excluded from both the MMX mesalazine and all-comparator subgroups. More than 10 % of patients with UC in the MMX mesalazine and 5-ASA comparator groups were receiving other types of medication at baseline, including acetaminophen, potassium chloride, hydrocodone, metronidazole, other mesalazines, ciprofloxacin, and sodium bicarbonate. The most common preindex medical conditions present in patients at baseline were gastrointestinal inflammation, diarrhea, abdominal pain, rectal hemorrhage, essential hypertension, and hematochezia, each occurring in >15% of patients in both the MMX mesalazine and comparator groups.

**Table 2:** Patient demographic and baseline characteristics

Patient characteristic	MMX mesalazine (*n* = 4,751)	All comparators (*n* = 10,176)	Total (*n* = 14,936)
Mean age (median), years	44.38 (44.0)	47.44 (47.0)	46.46 (46.0)
Age categories, *n* (%)			
18–19	130 (2.7)	187 (1.8)	318 (2.1)
20–29	740 (15.6)	1,367 (13.4)	2,108 (14.1)
30–39	1,031 (21.7)	1,829 (18.0)	2,862 (19.2)
40–49	1,075 (22.6)	2,172 (21.3)	3,249 (21.8)
50–59	1,013 (21.3)	2,307 (22.7)	3,322 (22.2)
60–69	532 (11.2)	1,451 (14.3)	1,984 (13.3)
70–79	164 (3.5)	534 (5.2)	698 (4.7)
≥80	66 (1.4)	329 (3.2)	395 (2.6)
Gender, *n* (%)			
Female	2,528 (53.2)	5,527 (54.3)	8,060 (54.0)
Male	2,223 (46.8)	4,649 (45.7)	6,876 (46.0)
Index date, *n* (%)			
Year 2008	1,589 (33.4)	3,958 (38.9)	5,549 (37.2)
Year 2009	1,953 (41.1)	3,608 (35.5)	5,566 (37.3)
Year 2010	1,209 (25.4)	2,610 (25.6)	3,821 (25.6)
Common baseline pharmacotherapy (>10% of patients), *n* (%)			
Acetaminophen	904 (19.0)	2,106 (20.7)	3,010 (20.2)
Potassium chloride	1,049 (22.1)	1,827 (18.0)	2,877 (19.3)
Hydrocodone	650 (13.7)	1,596 (15.7)	2,246 (15.0)
Metronidazole	627 (13.2)	1,536 (15.1)	2,165 (14.5)
Other mesalazine	857 (18.0)	1,035 (10.2)	1,893 (12.7)
Ciprofloxacin	546 (11.5)	1,290 (12.7)	1,839 (12.3)
Sodium bicarbonate	560 (11.8)	948 (9.3)	1,508 (10.1)
Common pre-index medical conditions (>10% of patients), *n* (%)			
Gastrointestinal inflammation	1,851 (39.0)	4,035 (39.7)	5,891 (39.4)
Diarrhoea	1,559 (32.8)	3,353 (33.0)	4,913 (32.9)
Abdominal pain	1,110 (23.4)	2,797 (27.5)	3,910 (26.2)
Rectal hemorrhage	1,111 (23.4)	2,131 (20.9)	3,245 (21.7)
Essential hypertension	742 (15.6)	2,047 (20.1)	2,790 (18.7)
Hematochezia	866 (18.2)	1,666 (16.4)	2,534 (17.0)
Gastrointestinal disorder	584 (12.3)	1,107 (10.9)	1,691 (11.3)
Hyperlipidemia	447 (9.4)	1,166 (11.5)	1,613 (10.8)
Hemorrhoids	483 (10.2)	1,007 (9.9)	1,491 (10.0)
Benign colonic neoplasm	485 (10.2)	976 (9.6)	1,463 (9.8)

### Crude incidence and relative risk of acute pancreatitis

The primary outcome measured was the occurrence of acute pancreatitis in patients receiving MMX mesalazine compared with those receiving comparator mesalazines. Results from this analysis are provided in Table 3. From study-drug index date through 30 days postdiscontinuation, 20 and 46 acute pancreatitis cases associated with a hospital/ER visit were identified for the MMX mesalazine and comparator groups, respectively, with 2,338 and 4,576 person-years of exposure, respectively (Table 3).

**Table 3:** Acute pancreatitis incidence rates (IR) and incident rate ratios (IRR; crude and adjusted)

Characteristic	MMX mesalazine	All comparators	IRR (95 % CI)^a^
Crude rates			
Events, *n*	20	46	
Person-time, years	2,338.2	4,576.1	
IR (95 % CI)^b^	8.55 (5.54–13.21)	10.05 (7.54–13.41)	0.85 (0.48–1.47)
Age categories, years IR (95 % CI)^b^			
18–29	20.03 (10.15–39.53)	29.70 (18.78–46.94)	0.67 (0.25–1.63)
30–39	10.68 (4.56–25.00)	10.27 (5.20–20.27)	1.04 (0.27–3.60)
40–49	5.52 (1.88–16.22)	4.32 (1.68–11.11)	1.28 (0.19–7.55)
≥50	4.32 (1.68–11.10)	7.06 (4.35–11.47)	0.61 (0.15–1.89)
Gender, IR (95 % CI)^b^			
Female	10.91 (6.37–18.66)	8.94 (5.84–13.66)	1.22 (0.56–2.55)
Male	6.11 (2.96–12.61)	11.23 (7.61–16.58)	0.54 (0.20–1.29)
Adjusted rates			
Events, *n*	20	21	
Person-time, years	2,220.07	1,958.90	
Propensity-matched IR (95 % CI)^b^	9.01 (5.81–13.96)	10.72 (6.99–16.44)	0.84 (0.46–1.55)
Adjusted 1 IR (95 % CI)^c^	4.40 (1.94–9.98)	5.62 (2.57–12.31)	0.78 (0.42–1.46)
Adjusted 2 IR (95 % CI)^d^	2.37 (0.91–6.15)	3.10 (1.24–7.75)	0.76 (0.41–1.43)
Age categories, years			
18–29			
Propensity-matched IR (95 % CI)^b^	21.43 (10.72–42.86)	26.77 (13.93–51.45)	0.80 (0.31–2.08)
Adjusted 1 IR (95 % CI)^c^	25.17 (10.50–60.33)	32.76 (15.15–70.83)	0.77 (0.29–2.01)
Adjusted 2 IR (95 % CI)^d^	22.77 (8.52–60.86)	30.66 (12.89–72.96)	0.74 (0.28–1.95)
30–39			
Propensity-matched IR (95 % CI)^b^	11.10 (4.62–26.67)	12.16 (5.06–29.22)	0.91 (0.26–3.15)
Adjusted 1 IR (95 % CI)^c^	9.06 (1.78–46.10)	10.89 (3.01–39.38)	0.83 (0.21–3.23)
Adjusted 2 IR (95 % CI)^d^	3.61 (0.42–30.63)	4.35 (0.78–24.29)	0.83 (0.20–3.44)
40–49			
Propensity-matched IR (95 % CI)^b^	5.80 (1.87–17.99)	4.80 (1.20–19.17)	1.21 (0.20–7.24)
Adjusted 1 IR (95 % CI)^c^	2.13 (0.26–17.66)	1.89 (0.20–17.76)	1.13 (0.18–6.99)
Adjusted 2 IR (95 % CI)^d^	1.42 (0.13–15.19)	1.25 (0.10–15.80)	1.13 (0.18–7.00)
≥50			
Propensity-matched IR (95 % CI)^b^	4.55 (1.71–12.12)	6.29 (2.62–15.12)	0.72 (0.19–2.69)
Adjusted 1 IR (95 % CI)^c^	3.56 (0.39–32.19)	4.60 (0.99–21.25)	0.77 (0.16–3.80)
Adjusted 2 IR (95 % CI)^d^	1.80 (0.17–19.15)	2.26 (0.37–13.86)	0.80 (0.16–3.97)
Gender			
Female			
Propensity-matched IR (95 % CI)^b^	11.52 (6.69–19.83)	6.95 (3.32–14.59)	1.66 (0.66–4.15)
Adjusted 1 IR (95 % CI)^c^	7.72 (3.05–19.55)	4.94 (1.81–13.51)	1.56 (0.61–4.00)
Adjusted 2 IR (95 % CI)^d^	3.58 (1.12–11.46)	2.35 (0.70–7.83)	1.52 (0.59–3.92)
Male			
Propensity-matched IR (95 % CI)^b^	6.41 (3.06–13.46)	14.70 (8.71–24.82)	0.44 (0.18–1.08)
Adjusted 1 IR (95 % CI)^c^	2.00 (0.54–7.45)	4.70 (1.43–15.38)	0.43 (0.17–1.07)
Adjusted 2 IR (95 % CI)^d^	1.05 (0.22–5.10)	2.52 (0.58–10.98)	0.42 (0.17–1.04)

*CI* confidence interval

^a^MMX mesalazine relative to all comparators

^b^Per 1000 person-years

^c^Adjustment 1, adjusted for age category, gender, and daily dose

^d^Adjustment 2, adjusted for age category, gender, daily dose, steroids, enalapril, viral herpes, furosemide, sulfasalazine, and post-surgery procedures

The overall crude IR [95 % confidence interval (CI)] of acute pancreatitis in the MMX mesalazine group was 8.55 (5.54–13.21) per 1,000 person-years compared with 10.05 (7.54–13.41) per 1,000 person-years in the all comparators group. In regards to relative risk in the overall population, the crude IRR (95 % CI) was 0.85 (0.48–1.47), suggesting that no difference in pancreatitis risk existed prior to adjustment between the MMX mesalazine and all comparator groups (Table 3). Among the comparator subgroups by 5-ASA release mechanism, crude pancreatitis IRs (95 % CI) were lowest in the azoreductase-dependent group [5.11 (0.90–28.92) per 1,000 person-years] and highest in the moisture-dependent group [10.88 (2.98–39.68) per 1,000 person-years].

Sensitivity analyses varying both the drug persistence gap and risk period between 15, 30, and 60 days had little influence on the overall incidence of acute pancreatitis in the MMX mesalazine group. Similarly, the IRRs of the MMX mesalazine and comparator group were relatively stable (range 0.78–0.89; data not shown).

### Adjusted incidence and relative risk of acute pancreatitis

Of the 4,751 patients in the MMX mesalazine group, 4,499 (94.7 %) patients were propensity-score matched to comparators. No pancreatitis events occurred in the unmatched MMX mesalazine group. After matching, 20 acute pancreatitis cases were identified in the MMX mesalazine group, and 21 cases were identified in the all comparators group. Prior to matching, propensity scores for the MMX mesalazine and the all-comparator groups were 0.341 and 0.305, respectively, reflecting a 12 % difference. After matching, the all-comparator group had a mean propensity score of 0.340, essentially unchanged for the MMX mesalazine group. Of the 4,499 MMX mesalazine and 4,499 comparator propensity-score–matched patients, 16 MMX mesalazine and 11 comparator patients were missing information needed to calculate average daily dose; thus, the following adjusted incidence analyses were conducted on 4,483 MMX mesalazine and 4,488 comparator patients.

The propensity-matched IR (95 % CI) of acute pancreatitis among patients on MMX mesalazine [9.01 (5.81–13.96) per 1,000 person-years] was comparable with that of comparator 5-ASA formulations [10.72 (6.99–16.44) per 1,000 person-years]. All propensity-score–matched analyses yielded comparable IRRs to the crude IRR. From the model that incorporated propensity-score matching only, the IRR (95 % CI) for MMX mesalazine relative to all comparators was 0.84 (0.46–1.55). Propensity-score–matched models further adjusted (age, gender, dose; Adjusted 1) and fully adjusted (steroids, enalapril, viral herpes, furosemide, sulfasalazine, and postsurgery procedures added; Adjusted 2) yielded IRRs of 0.78 (0.42–1.46) and 0.76 (0.41–1.43), respectively (Table 3). Comparisons between MMX mesalazine and comparator subgroups defined by 5-ASA release mechanism were not possible in the adjusted analyses due to the limited number of events.

### Prescribing patterns

MMX mesalazine users were almost always prescribed a medium average daily dose, whereas nearly one in five comparator drug users were prescribed a low daily dose. Average daily dose was entered into the two further adjusted models that estimated relative risk. Prescribing pattern data are presented in Supplementary Table 3.

## Discussion

The impetus for conducting this pharmacoepidemiology study was a signal of disproportionate reporting of postmarketing pancreatitis cases, suggesting a potentially elevated risk of pancreatitis for MMX mesalazine compared with other mesalazine formulations used to treat UC. Because spontaneous reporting data lack complete case ascertainment and defined patient populations in which to compute IRs and are thus subject to reporting biases, further evaluation of this possible relationship was warranted. The primary objective of this pharmacoepidemiology study was to formally evaluate whether the risk of pancreatitis among patients with UC using MMX mesalazine differed from the risk among similar patients using other branded orally administered mesalazine drugs. Results from this analysis demonstrated that the adjusted IRR was 0.76 (0.41–1.43), which suggests that the risk for pancreatitis is not elevated for MMX mesalazine. In estimating this relative risk, confounding factors controlled for in the pharmacoepidemiology study, but not in the reporting data, included the calendar period under comparison, age and gender of the patient, and dose, as well as baseline comorbidity and concomitant medication.

Acute pancreatitis is a multifactorial condition with many known causes or combination of causes, such as gallstones, alcohol use, hypertriglyceridemia, and >500 implicated medications [26]. Even for suspected drug-induced pancreatitis, it is proposed that drugs are most likely a trigger for pancreatitis in patients with other risk factors [27]. Therefore, given the nonspecificity of mesalazine as a cause of pancreatitis, and that this study was comparing MMX mesalazine to other mesalazine drugs to determine whether MMX mesalazine was associated with a further elevation in risk, it was extremely important to control for the multitude of competing risk factors, most prominently, concomitant medications. The spontaneous reports did not allow for this control, whereas in the pharmacoepidemiology study, some of these factors were used to adjust relative risk estimates.

The other prominent bias in the spontaneous reporting data for MMX mesalazine and pancreatitis was reporting bias for MMX mesalazine due to the Webber effect [28]. This bias was likely present due to the different lengths of time on the US market between MMX mesalazine (2007) and the comparator drugs controlled-release mesalazine (1993) and delayed-release mesalazine (1992). It has been well established that newer drugs are monitored more closely for adverse effects (AEs) and that those AEs are more likely to be reported than medications that have been in long-term use [29]. Whereas healthcare claims data sources are subject to selection biases related to the population recorded, there is no reporting bias affecting the recording of an adverse event. Thus, this pharmacoepidemiology study was an effective means for eliminating that hurdle, which was likely present in the spontaneous reporting data. 

Despite the methodological advantages of using a pharmacoepidemiologic study to evaluate a signal generated via spontaneous reporting, several limitations of this analysis still exist. Pancreatitis is a rare event, so despite the inclusion of >4,000 patients in each treatment group, adjusted effect estimates were somewhat imprecise. However, the fully adjusted model revealed an IRR point estimate of 0.76, with an upper bound of the 95 % CI of <1.5, which, if valid, suggests that any increase in risk is very likely to be small. Another potential limitation is that the accuracy of ICD-9 codes used to detect cases of acute pancreatitis may be questioned, as case status was not validated in hospital records. However, prior research has demonstrated that the ability to properly identify acute pancreatitis using administrative claims data is good; the ICD-9-CM 557.0 codes for acute pancreatitis have demonstrated high sensitivity (93 %) and good specificity (72 %) when recorded as the primary diagnosis [30]. The algorithm we used to define an acute pancreatitis event was similar, suggesting that diagnostic miscoding was likely minimal. Finally, alcohol consumption is an important covariate associated with the risk of pancreatitis [31]. However, because alcohol consumption may not be validly or reliably recorded in healthcare claims data sources [32], it was excluded from our analysis so as not to introduce bias due to differences in ascertainment. Because alcohol consumption is not a contraindication for MMX mesalazine or comparator drugs, it was assumed that the groups were fairly balanced on this risk factor; however, without data, this assumption cannot be confirmed.

## Conclusions

The signal of disproportionate reporting of pancreatitis for patients treated with MMX mesalazine in relation to other mesalazine medications was not confirmed by a formal pharmacoepidemiology study. By using healthcare claims data (that capture healthcare encounters regardless of whether they were reported as adverse events), the study eliminated potential spontaneous reporting bias due to time between launch of MMX mesalazine relative to comparator drugs. In addition, the study controlled confounding due to risk factors for pancreatitis (e.g., disease under treatment, concomitant medications, and comorbid conditions) by propensity-score matching and multivariate modelling. These findings demonstrate the value of pharmacoepidemiology studies for evaluating a drug’s postmarket safety profile when confronted with spontaneous reporting data suggestive of a safety issue.

## Electronic supplementary material

Below is the link to the electronic supplementary material.

Supplementary Table 1 (DOCX 12 kb)

Supplementary Table 2 (DOCX 12 kb)

Supplementary Table 3 (DOCX 16 kb)

## Footnotes

1 MMX Multi Matrix System® and MMX® are registered trademarks of Cosmo Technologies Ltd., Wicklow, Ireland.

## References

[1] Ahmad SR (2003). Adverse drug event monitoring at the Food and Drug Administration. J Gen Intern Med.

[2] U.S. Department of Health and Human Services Food and Drug Administration Center for Drug Evaluation and Research (CDER) Center for Biologics Evaluation and Research (CBER (2005) Guidance for Industry Good Pharmacovigilance Practices and Pharmacoepidemiologic Assessment. Available at: http://www.fda.gov/downloads/regulatoryinformation/guidances/ucm126834.pdf. Accessed 16 Nov 2012

[3] Strom BL (2006). How the US drug safety system should be changed. JAMA.

[4] Kornbluth A, Sachar DB (2010). Ulcerative colitis practice guidelines in adults: American College of Gastroenterology, Practice Parameters Committee. Am J Gastroenterol.

[5] D'Haens G, Sandborn WJ, Barrett K, Hodgson I, Streck P (2012). Once-daily MMX(R) mesalamine for endoscopic maintenance of remission of ulcerative colitis. Am J Gastroenterol.

[6] Kane S, Katz S, Jamal MM, Safdi M, Dolin B, Solomon D, Palmen M, Barrett K (2012). Strategies in maintenance for patients receiving long-term therapy (SIMPLE): a study of MMX mesalamine for the long-term maintenance of quiescent ulcerative colitis. Inflamm Bowel Dis.

[7] Lichtenstein GR, Kamm MA, Sandborn WJ, Lyne A, Joseph RE (2008). MMX mesalazine for the induction of remission of mild-to-moderately active ulcerative colitis: efficacy and tolerability in specific patient subpopulations. Aliment Pharmacol Ther.

[8] Sandborn WJ, Kamm MA, Lichtenstein GR, Lyne A, Butler T, Joseph RE (2007). MMX Multi Matrix System mesalazine for the induction of remission in patients with mild-to-moderate ulcerative colitis: a combined analysis of two randomized, double-blind, placebo-controlled trials. Aliment Pharmacol Ther.

[9] Banks PA, Bollen TL, Dervenis C, Gooszen HG, Johnson CD, Sarr MG, Tsiotos GG, Vege SS (2013). Classification of acute pancreatitis–2012: revision of the Atlanta classification and definitions by international consensus. Gut.

[10] Sandzen B, Rosenmuller M, Haapamaki MM, Nilsson E, Stenlund HC, Oman M (2009) First attack of acute pancreatitis in Sweden 1. BMC Gastroenterol 918 10.1186/1471-230X-9-18 PMC2669478 19265519

[11] Barthet M (2009). Acute pancreatitis: an emerging presentation for autoimmune pancreatitis in patients with inflammatory bowel disease. Gastroenterol Hepatol (N Y).

[12] Lankisch PG, Droge M, Gottesleben F (1995). Drug induced acute pancreatitis: incidence and severity. Gut.

[13] LIALDA (2011). LIALDA® (mesalamine) delayed-release tablets, for oral use [package insert].

[14] FDA Adverse Event Reporting System (2013) FDA Adverse Event Reporting System (FAERS) (formerly AERS). Available at: http://www.fda.gov/Drugs/GuidanceComplianceRegulatoryInformation/Surveillance/AdverseDrugEffects/default.htm

[15] Robb MA, Racoosin JA, Sherman RE, Gross TP, Ball R, Reichman ME, Midthun K, Woodcock J (2012). The US Food and Drug Administration's Sentinel Initiative: expanding the horizons of medical product safety. Pharmacoepidemiol Drug Saf.

[16] International Society for Pharmacoepidemiology (1998) Data Privacy, Medical Record Confidentiality, and Research in the Interest of Public Health. Available at: http://www.pharmacoepi.org/resources/privacy.cfm 10.1002/(SICI)1099-1557(199907)8:4<247::AID-PDS432>3.0.CO;2-O 15073916

[17] Dore DD, Chaudhry S, Hoffman C, Seeger JD (2011). Stratum-specific positive predictive values of claims for acute pancreatitis among commercial health insurance plan enrollees with diabetes mellitus. Pharmacoepidemiol Drug Saf.

[18] Eland IA, Sturkenboom MJ, Wilson JH, Stricker BH (2000). Incidence and mortality of acute pancreatitis between 1985 and 1995. Scand J Gastroenterol.

[19] Kane S, Shaya F (2008). Medication non-adherence is associated with increased medical health care costs. Dig Dis Sci.

[20] Kane SV, Accortt NA, Magowan S, Brixner D (2009). Predictors of persistence with 5-aminosalicylic acid therapy for ulcerative colitis. Aliment Pharmacol Ther.

[21] Kane SV, Sumner M, Solomon D, Jenkins M (2011). Twelve-month persistency with oral 5-aminosalicylic Acid therapy for ulcerative colitis: results from a large pharmacy prescriptions database. Dig Dis Sci.

[22] Kappelman MD, Rifas-Shiman SL, Porter CQ, Ollendorf DA, Sandler RS, Galanko JA, Finkelstein JA (2008). Direct health care costs of Crohn's disease and ulcerative colitis in US children and adults. Gastroenterology.

[23] Frossard JL, Steer ML, Pastor CM (2008). Acute pancreatitis. Lancet.

[24] Riley SA (1998). What dose of 5-aminosalicylic acid (mesalazine) in ulcerative colitis?. Gut.

[25] Pruitt R, Hanson J, Safdi M, Wruble L, Hardi R, Johanson J, Koval G, Riff D, Winston B, Cross A, Doty P, Johnson LK (2002). Balsalazide is superior to mesalamine in the time to improvement of signs and symptoms of acute mild-to-moderate ulcerative colitis. Am J Gastroenterol.

[26] Vinklerova I, Prochazka M, Prochazka V, Urbanek K (2010). Incidence, severity, and etiology of drug-induced acute pancreatitis. Dig Dis Sci.

[27] Spanier BW, Tuynman HA, van der Hulst RW, Dijkgraaf MG, Bruno MJ (2011). Acute pancreatitis and concomitant use of pancreatitis-associated drugs. Am J Gastroenterol.

[28] Hartnell NR, Wilson JP (2004). Replication of the Weber effect using postmarketing adverse event reports voluntarily submitted to the United States Food and Drug Administration. Pharmacotherapy.

[29] Nitsche C, Maertin S, Scheiber J, Ritter CA, Lerch MM, Mayerle J (2012). Drug-induced pancreatitis. Curr Gastroenterol Rep.

[30] Yadav D, Dhir R (2006) How accurate are ICD-9 codes for acute (Ap) and chronic (Cp) pancreatitis?-A large VA hospital experience. Pancreas 33(4)

[31] Yadav D, Lowenfels AB (2006). Trends in the epidemiology of the first attack of acute pancreatitis: a systematic review. Pancreas.

[32] Fowles JB, Fowler EJ, Craft C (1998). Validation of claims diagnoses and self-reported conditions compared with medical records for selected chronic diseases. J Ambul Care Manag.

