# Supplementary material for: Role of pharmacoepidemiology studies in addressing pharmacovigilance questions: a case example of pancreatitis risk among ulcerative colitis patients using mesalazine
Source: Eur J Clin Pharmacol. 2014 Mar 11;70(6):709–17. doi: 10.1007/s00228-014-1660-7 (PMC4025187; doi:10.1007/s00228-014-1660-7)
Supplement: Supplementary file 1 — (DOCX 12 kb) [file 228_2014_1660_MOESM1_ESM.docx]

**Supplementary Table 1. Eligibility Criteria**

| *Inclusion Criteria* |
| --- |
| - Adult patients (≥18 years) with a recorded diagnosis of UC (ICD-9 556, 556.0, 556.1, 556.5, 556.6, 556.8, and 556.9) within 6 months before receiving 1 of the study drugs (6-month baseline period) - New users of MMX mesalazine or a comparator 5-ASA UC treatment - Initial prescription of study drug between January 1, 2008 and December 31, 2010 (index date)*   *Although MMX mesalazine was initially available in March 2007, the period of MMX mesalazine uptake prior to study start was excluded because patients prescribed newly available pharmacotherapies (ie, “early adopters”) often represent a group where patient-, prescriber-, or payer-related factors may result in a biased channelling of patients to 1 treatment versus another |
| *Exclusion Criteria* |
| - Any prescription for MMX mesalazine or a comparator drug at any time before the index date - Any history of pancreatitis (ICD-9: 577.0-577.1) or Crohn’s disease (ICD-9: 555.x) during baseline - Surgery with colectomy during baseline or follow-up were also excluded, as this was an indication of severe UC, which was itself an exclusion from this study |

UC, ulcerative colitis; ICD, International Classification of Diseases; 5-ASA, 5-aminosalicylic acid.
