# Supplementary material for: Role of pharmacoepidemiology studies in addressing pharmacovigilance questions: a case example of pancreatitis risk among ulcerative colitis patients using mesalazine
Source: Eur J Clin Pharmacol. 2014 Mar 11;70(6):709–17. doi: 10.1007/s00228-014-1660-7 (PMC4025187; doi:10.1007/s00228-014-1660-7)
Supplement: Supplementary file 2 — (DOCX 12 kb) [file 228_2014_1660_MOESM2_ESM.docx]

**Supplementary Table 2. Propensity Score Methodology**

| *Propensity scores were constructed within the SÆfetyWorks software through a 2-phase process* |
| --- |
| *Phase 1*   - All eligible patients identified and stratified by age, gender, calendar year of index date, and exposure to MMX mesalazine or comparator drug - All patients also described by a list of thousands of binary covariates, corresponding to the presence or absence of the administration of a drug, or the occurrence of a medical condition or procedure occurring during the 6-month baseline period - Among covariates, candidate variables for the propensity model were determined to be those with crude odds ratio (OR) point estimates of >1.1 - Covariates also screened for collinearity; when collinear variables were identified, the variable with weaker association with the treatment choice was excluded |
| *Phase 2*   - Conducted from a logistic regression model, with drug exposure (MMX mesalazine vs comparator) as the outcome variable and the covariates selected in phase 1 as predictors - Age, gender, and calendar year forced into the model - All covariates included in the propensity score model required to be present in ≥60 patients (MMX mesalazine + comparators), have an OR ≥1.15, and have a lower bound of the 95% confidence interval of ≥0.99 |
