# Supplementary material for: Role of pharmacoepidemiology studies in addressing pharmacovigilance questions: a case example of pancreatitis risk among ulcerative colitis patients using mesalazine
Source: Eur J Clin Pharmacol. 2014 Mar 11;70(6):709–17. doi: 10.1007/s00228-014-1660-7 (PMC4025187; doi:10.1007/s00228-014-1660-7)
Supplement: Supplementary file 3 — (DOCX 16 kb) [file 228_2014_1660_MOESM3_ESM.docx]

**Supplemental Table 3. Prescribing Patterns of Propensity Matched Patients**

| Dose characteristic |  | MMX mesalazine (n = 4483) | All comparators (n = 4488) | Total  (n = 8971) |
| --- | --- | --- | --- | --- |
| Supply, days |  |  |  |  |
| Mean |  | 162.07 | 136.68 | 149.36 |
| Median |  | 90 | 90 | 90 |
| Fill quantity |  |  |  |  |
| Mean |  | 523.74 | 627.02 | 575.41 |
| Median |  | 240 | 360 | 360 |
| Tablets/pills/capsules per day |  |  |  |  |
| Mean |  | 3.72 | 5.61 | 4.67 |
| Median |  | 4 | 6 | 4 |
| Medication strength, mg |  |  |  |  |
| Mean |  | 4469.68 | 2421.13 | 3444.83 |
| Median |  | 4800 | 2400 | 2639 |
| Total average daily dose, n (%) |  |  |  |  |
| Low (<1500 mg/day) |  | 2 (0.04) | 864 (19.25) | 866 (9.65) |
| Medium (1500-4800 mg/day) |  | 4258 (94.98) | 3509 (78.19) | 7767 (86.58) |
| High (>4800 mg/day) |  | 223 (4.97) | 115 (2.56) | 338 (3.77) |
